# An Adrenocortical Carcinoma Associated with Non-Islet Cell Tumor Hypoglycemia and Aberrant ACTH Production

**DOI:** 10.1155/2020/2025631

**Published:** 2020-03-09

**Authors:** M. D. S. A. Dilrukshi, A. W. Wickramarachchi, D. D. K. Abeyaratne, Brian Shine, Bahram Jafar-Mohammadi, N. P. Somasundaram

**Affiliations:** ^1^Diabetes and Endocrinology Unit of National Hospital of Sri Lanka, Colombo 10, Sri Lanka; ^2^National Hospital of Sri Lanka, Colombo, Sri Lanka; ^3^Clinical Biochemistry, John Radcliffe Hospital, Oxford OX3 9DU, UK; ^4^Oxford Centre for Diabetes, Endocrinology and Metabolism, Churchill Hospital, Oxford University Hospitals NHS Foundation Trust, Oxford, UK

## Abstract

*Introduction*. Adrenocortical carcinomas (ACCs) are infrequently reported to present with severe hypoglycemia syndrome resulting from the secretion of insulin-like growth factor II (IGF-II) by tumor cells. Adrenocorticotropic hormone- (ACTH) independent hypercortisolism is the norm of hormonally active ACCs, but aberrant ACTH production by tumor cells can theoretically cause ACTH-dependent hypercortisolism. The purpose of this report was to present a case of an ACC manifested with the co-occurrence of two extremely rare presentations. *Case Description*. We present a rare case of a 43-year-old male patient admitted with recurrent episodes of severe non-ketotic and non-insulin-mediated hypoglycemia due to IGF-II mediated disease and ACTH-dependent Cushing's syndrome. He was diagnosed with a diffusely disseminated adrenocortical carcinoma with immunohistochemistry of tumor cells showing focal ACTH immunostain positivity. *Conclusion*. Non-islet cell tumor hypoglycemia and ACTH-dependent Cushing's syndrome are extremely rare presentations of an ACC, and co-occurrence of these entities in a single patient is never reported in the literature.

## 1. Introduction

Adrenocortical carcinomas (ACCs) are rare but aggressive tumors which show an incidence of 1-2 cases per million populations per year [1]. Non-islet cell tumor hypoglycemia (NICTH) is a rare paraneoplastic syndrome of ACCs which was described first by Anderson in 1930 and a handful of cases reported thereafter [2]. Cushing's syndrome due to ectopic adrenocorticotropic hormone (ACTH) secretion was first described by Hurst Brown in 1928 [3] in association with bronchial carcinoma. Though non-ACTH-dependent hypercortisolism is the norm of adrenal malignancies, ectopic ACTH secretion by adrenal tumor cells can theoretically result in ACTH-dependent Cushing's syndrome. To the best of our knowledge, the co-occurrence of NICTH and ACTH-dependent hypercortisolism due to aberrant ACTH producing tumor cells is never reported in the medical literature. We report a unique case of a 43-year-old man with disseminated adrenocortical carcinoma presenting with these two rare disease manifestations coincidentally.

## 2. Case Report

A 43-year-old Sri Lankan male presented to a tertiary care hospital of Sri Lanka (SL) in January 2019 with recurrent episodes of early morning dizzy spells of six-month duration and a history of resistant hypertension and intra-abdominal malignancy for which he received several types of treatment abroad and locally until 3 months before.

He started to experience recurrent episodes of early morning dizzy spells which were associated with excessive hunger, sweating, and palpitations that were resolving with a sugary drink or food in July 2018. These episodes were confirmed to be hypoglycemia at a local hospital with positive Whipple's triad while awaiting surgery for the aforementioned malignancy. There was some improvement of these symptoms after resection of the tumor; hence, no further evaluation was performed. However, hypoglycemia symptoms recurred in September 2019 and gradually progressed thereafter with increasing frequency and severity, up until 2 weeks, when he started to develop episodes of disorientation and confusion, which prompted him to seek medical attention. There were no episodes of coma or seizures to report, and he denied symptoms of overt liver or renal failure apart from the loss of appetite. He neither had features of clinical depression nor history to suggest abuse of oral hypoglycemic agents, insulin, recreational drugs, or alcohol. He was off from any medication over 3 months before the admission.

His past medical history revealed poorly controlled hypertension since mid-2017 which was not properly controlled as he went for employment abroad. According to his medical records, he admitted to a hospital abroad in January 2018, with subacute onset moderate to severe retrosternal chest pain with difficulty in breathing. Additionally, he complained of progressive abdominal distension, nonspecific abdominal pain, significant loss of appetite, loss of weight of 2 months, and chronic watery stools without blood or mucus discharge of similar duration. He also had recurrent nonspecific headaches and palpitations but no episodes of flushing. Examination findings on admission recorded pulse rate (PR) of 96 beats per min with a blood pressure (BP) of 170/100 mmHg. Laboratory workup showed hypokalemia (K^+^ 2.8 mmol/l), normal blood counts, renal, and liver functions. He was noted to have heart failure with an ejection fraction of 38% with a concentric left ventricular hypertrophy. His abdominal ultrasonography and subsequent whole-body PET/CT had shown a left suprarenal mass of 22 cm × 13 cm × 23 cm in size with coeliac and para-aortic lymphadenopathy (maximum diameter of 3 cm), and hepatic, lung parenchymal, and subpleural metastasis. Biopsy of abdominal mass had shown tumor cells with a high degree of anaplasia, and immunohistochemistry was compatible with probable adrenocortical malignancy. He had received 3 cycles of chemotherapy according to the records, but no details were mentioned regarding the types or names of medications.

He had received 5 months of Ayurveda treatment after returning to SL before admitting to a local hospital in July 2018, with an uncontrolled blood pressure of 180/90 mmHg. Apart from the recurrent episodes of early morning hypoglycemia, he was otherwise well with normal systemic review and had stable weight. Basic Lab workup was normal except for mild anemia, hypokalemia, and mildly elevated transaminase levels (Table 1).

**Figure 1:**
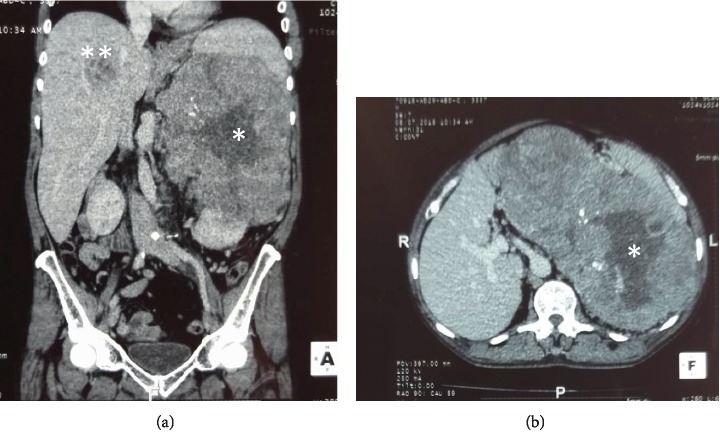
Contrast-enhanced CT abdomen, chest, and pelvis: (a) coronal image and (b) axial image showing heterogeneously enhancing left suprarenal mass (21 × 18 × 24 cm)^*∗*^ with central necrosis and foci of calcifications. Focal liver metastasis^*∗∗*^ is also seen.

Contrast-enhanced CT (CECT) scan of the chest, abdomen, and pelvis showed previously detected heterogeneously enhancing left suprarenal mass with central necrosis and foci of calcifications without significant interval change in size. There were multiple liver, lymph node, and pulmonary metastasis to note (Figure 1).

He underwent resection of abdominal mass in July 2018 and made an uneventful and complete recovery after the surgery with improvement in symptoms of hypoglycemia. However, histology of the tumor cells showed a high degree of anaplasia and basic immunohistochemistry pattern was more in favor of a malignant pheochromocytoma. Nevertheless, the patient defaulted from follow-up diverting back to Ayurveda treatment without waiting for further evaluation and management.

He had no significant family history of malignancy or chronic diseases to note. He was a social drinker and a non-smoker.

On examination at our center, he was seemingly well with a BMI of 25.2 kg/m^2^. His PR was 96 beats per minute, and BP was 180/90 mmHg. He had large tender hepatomegaly and a well-healed midline abdominal scar. The rest of the clinical examination was unremarkable with no edema, generalized pigmentation, or other discriminatory features of Cushing's syndrome except for significant proximal myopathy.

During the hospital stay, his vital parameters were stable, and the lowest recorded blood sugar level was 15 mg/dL with symptoms. His laboratory workup revealed neutrophil leukocytosis, anemia, mildly elevated transaminases, and normal renal functions (Table 2).

Further biochemical evaluation of persistently low blood sugar was favoring non-ketotic and non-insulin-dependent hypoglycemia (Table 3). Considering the high probability of IGF-II-mediated hypoglycemia, we sent a serum sample to a reference laboratory in the United Kingdom since the facility to perform IGF-II is not available in SL. Lab results showed serum IGF-II level of 108.8 nmol/L and markedly elevated IGF-II : IGF-I ratio favoring non-islet cell tumor hypoglycemia (NICTH).

**Figure 2:**
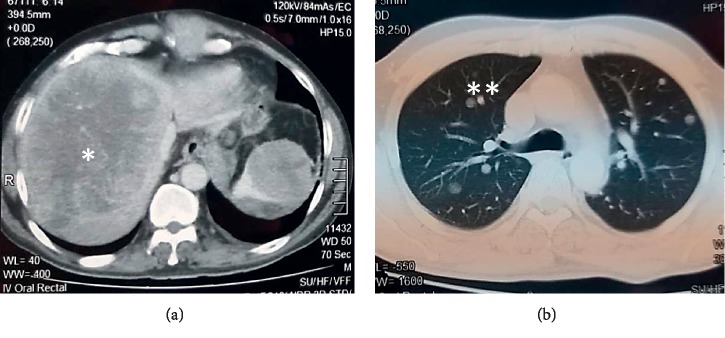
CECT chest and abdomen (axial image): large liver metastasis^*∗*^ (largest: 12 × 11 cm in segment VII and VIII) and lung metastases^*∗∗*^.

Moreover, CECT chest, abdomen, and pelvis showed recurrence of the previously detected tumor (42  mm × 37  mm, 85 × 68 mm and 73 × 86 mm size tumor masses intra-abdominally) with multiple liver (largest of the metastases being 12 × 11 cm in size in segment VII and VIII), renal (40 × 62 mm), and lung metastases (Figure 2). But there were no metastases in the right adrenal gland to note.

Adrenal hormone analysis was performed as the clinical picture was suggestive of a biologically active tumor, in addition to the dilemma created by the initial histology report (Table 4). The hormone profile revealed Cushing's syndrome with nonsuppressed ACTH levels favoring ACTH-dependent Cushing's syndrome. Additionally, adrenal androgens were also elevated with normal urine metanephrines favoring a tumor of adrenocortical origin.

**Figure 3:**
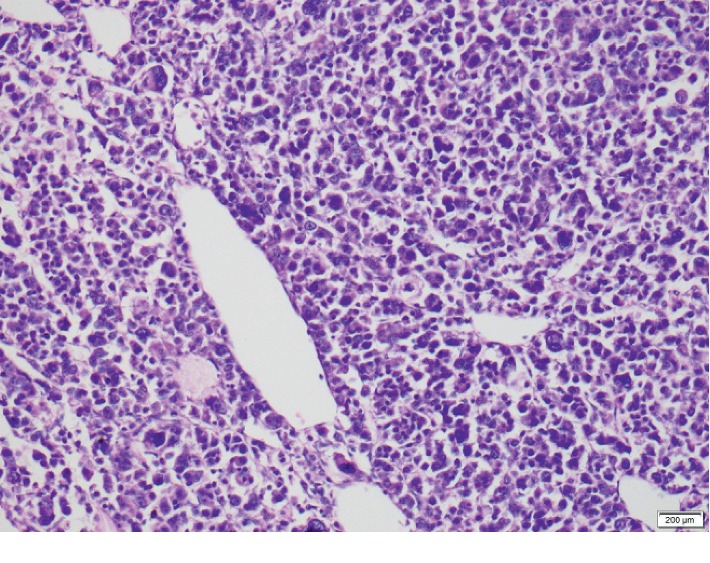
H&E stain (high power): solid sheets and nests of tumor cells, containing pleomorphic vesicular nuclei with coarse chromatin and eosinophilic cytoplasm.

We reassessed tumor tissues in parallel to the above evaluation with extended immunohistochemistry (IHC) profiles. Histology (Figure 3) showed an encapsulated multinodular tumor composed of solid sheets and nests of tumor cells with thick fibrous bands separating them, containing pleomorphic vesicular nuclei with coarse chromatin and eosinophilic cytoplasm. Mitoses were present >30/50 per high power field including atypical forms. There were areas of high nuclear pleomorphism with prominent nucleoli and bizarre multinucleated cells (Fuhrman grade 4). Tumor necrosis and vascular invasion were present. However, clear cell areas were not seen in the examined section.

**Figure 4:**
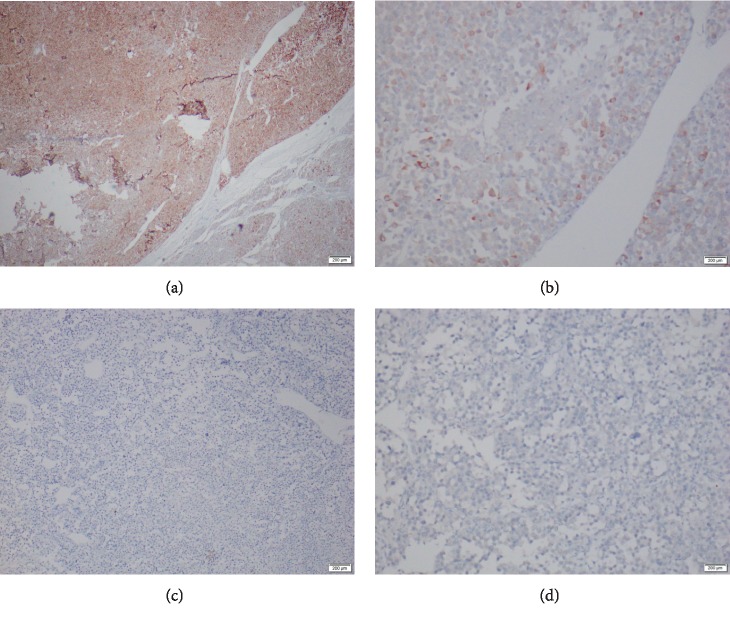
Immunohistochemical staining: (a) synaptophysin stain (patchy positivity), (b) inhibin stain (focal cytoplasmic positivity), (c) Chromogranin stain (negative), and (d) Pan CK stain (negative).

The IHC staining profile (Figure 4) showed patchy positivity for synaptophysin and focal cytoplasmic positivity for inhibin. Tumor cells were immunonegative for chromogranin, melan A, and pan CK. Overall, the negativity of cytokeratin and chromogranin with the focal positivity of inhibin was in favor of a tumor of adrenal cortical origin. The absence of clear cell areas also favors against pheochromocytoma. The sheet-like diffuse growth pattern, areas with a high nuclear grade, high mitotic rate with atypical forms, absence of clear cells, presence of tumor necrosis, venous invasion, and capsular invasion are correlating with the malignant behavior of the adrenocortical tumor with Weiss criteria of 8/9 [4].

**Figure 5:**
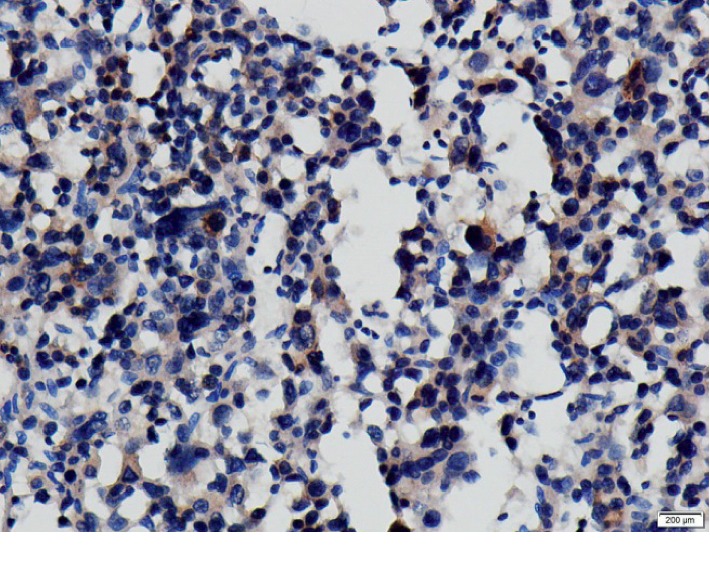
ACTH Immunostaining: few scattered tumor cells showing granular cytoplasmic positivity.

As the patient had ACTH-dependent Cushing's syndrome with a theoretical possibility of tumor cells secreting ACTH, we included ACTH immunostain to the IHC profile. Tumor cells showed granular cytoplasmic positivity for ACTH favoring aberrant ACTH production by ACC cells (Figure 5).

Alleviation of the troublesome hypoglycemia to improve quality of life was a priority of this patient's management. He was offered frequent meals and intermittent oral dextrose but failing that commenced on continuous intravenous dextrose infusions with frequent monitoring of blood sugar. Oral diazoxide seemed not effective. Subsequently, the patient was commenced on subcutaneous recombinant growth hormone (rhGH) 3 mg/daily and rapidly increased up to 4 mg/daily for which he showed a significant reduction of severity and frequency of hypoglycemia, hence could limit dextrose infusions. After extensive discussions with the oncologist, patient, and the family, he was opted for palliative chemotherapy believing that reduction of tumor burden to some degree would further help to alleviate symptoms of hypoglycemia. Unfortunately, the patient succumbed to the illness while receiving palliative chemotherapy.

## 3. Discussion

This patient was diagnosed to have an aggressive, functional adrenocortical carcinoma with diffuse metastasis, which was complicated with paraneoplastic severe NICTH and ACTH-dependent Cushing's syndrome resulting from aberrant ACTH secretion.

This patient had hypercortisolism, hyperandrogenemia and negative urine metanephrines in the presence of disseminated adrenal malignancy favoring a tumor of adrenocortical origin. But there was a misclassification of the tumor in the initial histology report. The considerable difficulty associated with differentiation between adrenocortical (ACC) and medullary (pheochromocytoma) tumor with a high degree of anaplasia was previously noted in the medical literature [5]. Even though comparatively higher nuclear pleomorphism and the presence of hyaline globules in the cytoplasm are characteristics of pheochromocytoma those are not specific for the tumor [6]. IHC studies are useful ancillary tests to the differentiation of tumors in this context. Immunopositivity for inhibin is suggestive of an adrenocortical tumor, while strong and diffuse positivity of chromogranin A is expected in pheochromocytoma [7]. The IHC profile of our patient was suggestive of the diagnosis of an ACC which was further strengthened by supportive clinical and laboratory data.

NICTH is a rare paraneoplastic syndrome that is only second to insulinoma as a cause for tumor-induced hypoglycemia [8]. This is commonly seen in tumors of mesenchymal and epithelial cell origin, and approximately 290 cases reported related to this topic so far in the medical literature. NICTH associated with adrenocortical carcinoma is even rarer [9]. The major pathophysiological mechanism behind this syndrome is tumor secretion of abnormal high molecular weight or “big” IGF-II (molecular weight of “big” IGF-II is 10-20 kDa as compared to normal IGF-II which is a 7.5 kDa peptide) which has insulin-like activity [9]. Tumors with aberrant IGF-II gene transcription and gene expression produce this big IGF-II due to abnormal processing. Normal IGF-II molecules circulate as a ternary complex after binding with IGF-binding protein-3 (IGFBP-3) and acid-labile subunit (ALS) thus are incapable of traversing epithelial barriers to bind to insulin receptors. In NICTH, elevated total IGF-II leads to a greater concentration of free form as well as abnormal high molecular weight precursor big IGF-II cannot be bound to ALS due to steric hindrance and thus stay in binary complex binding to IGFBP-3 which could easily go through vascular barriers to act on insulin receptors exerting hypoglycemic effects. Additionally, the activity of IGF-II suppresses both insulin and growth hormone (GH) with resultant low insulin and IGF-I. Low GH also leads to low ALS and IGFBP-3, further elevating the free IGF-II forms [9]. In this condition, serum levels of IGF-II may be elevated or normal and levels of IGF-II precursors are often elevated, and hence elevated IGF-II : IGF-I ratio is used for the diagnosis purpose. The normal molar ratio of IGF-II : IGF-I in plasma is about 3 : 1 and in the presence of recurrent hypoglycemia ratios of 10 : 1 are virtually diagnostic for IGF-II-mediated hypoglycemia [10]. Our patient had significantly elevated IGF-II : IGF-I ratio of 38.9 (<10) in the presence of non-ketotic non-insulin-dependent hypoglycemia and disseminated ACC, favoring this diagnosis. IHC staining of tumor cells for IGF-II could further strengthen the diagnosis in this context, which we could not perform due to financial limitations and the unavailability of the test locally.

Definitive management of ACC is the mainstay of therapy for NICTH after the acute management of hypoglycemia, which seemed impossible to achieve with the extensive tumor burden of this patient. Glucocorticoids [9, 11], glucagon [9, 12], and rhGH [1] were reported in the literature [9] as possible therapeutic options for NICTH. Our patient was commenced on a supraphysiological dose of recombinant growth hormone which helped to alleviate hypoglycemia for a considerable degree when combined with frequent small meals and oral/IV dextrose.

Furthermore, non-ACTH-dependent Cushing's syndrome is the norm of adrenal-mediated Cushing syndrome commonly seen in association with hormonally active ACC. However, our patient had biochemically confirmed Cushing's syndrome with non-suppressed serum ACTH levels. ACCs were never reported to be associated with ectopic ACTH production in the medical literature thus far. But, we hypothesized that this could be a possibility since there was no obvious clinical or imaging evidence to suggest pituitary disease or other ectopic focus. This hypothesis was proved by the immunopositivity of tumor cells for ACTH, indicating aberrant ACTH production.

## 4. Conclusion

Non-islet cell tumor hypoglycemia is an extremely rare presentation of ACCs. Though non-ACTH-dependent hypercortisolism is the norm of ACC, it can be associated with ACTH-dependent Cushing's syndrome with aberrant ACTH production by tumor cells. To the best of our knowledge, the co-occurrence of these presentations associated with ACC is never reported in the literature. Increased level of suspicion and broad-minded approach in the evaluation of this type of rare endocrine malignancies would help in diagnosing its rare presentations.

## Figures and Tables

**Table 1:** Basic laboratory data performed at the local hospital.

Test	Result	Reference range
Hemoglobin	10.9 g/L	13–17 g/L
FBS	78.8 mg/dl	70–99 mg/dl
Na^+^	137 mmol/L	136–146 mmol/L
K^+^	3.2 mmol/L	3.5–5.1 mmol/L
Serum creatinine	0.48 mg/dl	0.7–1.2 mg/dl
AST	110	5–34 U/L
ALT	40	0–35 U/L

**Table 2:** Basic laboratory data performed at the tertiary care hospital.

Test	Result	Reference range
WBC	14.72 × 10^3^	4–10 × 10^3^/*μ*L
Neutrophils	11.28 × 10^3^	2–7 × 10^3^/*μ*L
Hemoglobin	9.3	11–16 g/dL
AST	127	5–34 U/L
ALT	43	0–35 U/L
Serum creatinine	56	80–115 *μ*mol/L
Na^+^	140	136–145 mmol/L
K^+^	3.4	3.5–5.1 mmol/L
PT	13.6 seconds	11–15 seconds

**Table 3:** Evaluation of hypoglycemia performed at the tertiary care hospital.

Test	Result	Reference range
Random blood sugar	35 mg/dl	80–140 mg/dl
Urine/serum ketone bodies	Negative	—
Serum insulin (when random blood sugar of 35 mg/dl)	<0.5 mU/L	2.9–25 mU/L
C-peptide	0.09 ng/ml	0.61–3.85 ng/ml
IGF-I	2.8 nmol/L	8.5–27.3 nmol/L
IGF-II	108.8 nmol/L	—
IGF II : IGF-I ratio	38.9	<10

**Table 4:** Hormone profile performed at the tertiary care hospital.

Test	Result	Reference range
Random cortisol (9am)	>1000 nmol/L	138–635 nmol/L
Serum ACTH (9am)	40.3 pg/ml	7–50 pg/ml
ODST	748.5 nmol/l	<50 nmol/L
LDDST	668 nmol/L	<50 nmol/L
Total testosterone	595 ng/dl	300–1000 ng/dL
Androstenedione	>10 ng/ml	0.7–3.6 ng/ml
DHEAS	>40.71 *μ*mol/l	3.8–13.1 *μ*mol/l
24 hr urinary VMA	8.5 mg/24h	1–11 mg/24 h
24 hr urinary fractionated metanephrine	0.17 mg/24 hours	<1 mg/24 hours
